# High resolution mapping of modified DNA nucleobases using excision repair enzymes

**DOI:** 10.1101/gr.174052.114

**Published:** 2014-09

**Authors:** D. Suzi Bryan, Monica Ransom, Biniam Adane, Kerri York, Jay R. Hesselberth

**Affiliations:** Department of Biochemistry and Molecular Genetics, Program in Molecular Biology, University of Colorado School of Medicine, Aurora, Colorado 80045, USA

## Abstract

The incorporation and creation of modified nucleobases in DNA have profound effects on genome function. We describe methods for mapping positions and local content of modified DNA nucleobases in genomic DNA. We combined in vitro nucleobase excision with massively parallel DNA sequencing (Excision-seq) to determine the locations of modified nucleobases in genomic DNA. We applied the Excision-seq method to map uracil in *E. coli* and budding yeast and discovered significant variation in uracil content, wherein uracil is excluded from the earliest and latest replicating regions of the genome, possibly driven by changes in nucleotide pool composition. We also used Excision-seq to identify sites of pyrimidine dimer formation induced by UV light exposure, where the method could distinguish between sites of cyclobutane and 6-4 photoproduct formation. These UV mapping data enabled analysis of local sequence bias around pyrimidine dimers and suggested a preference for an adenosine downstream from 6-4 photoproducts. The Excision-seq method is broadly applicable for high precision, genome-wide mapping of modified nucleobases with cognate repair enzymes.

Many different modifications of the four primary DNA nucleobases expand the chemical diversity of DNA and have profound effects on genome function. Intrinsic modifications (e.g., 5-methylcytosine and uracil) are integral to genetic and epigenetic regulation. Extrinsic modifications (e.g., pyrimidine dimers and nucleobase oxidation) arise from environmental exposures and can initiate aberrant cell growth or death. A detailed understanding of intrinsic and extrinsic nucleobase modification is necessary for a complete view of genetic and epigenetic regulation, but a global picture of how nucleobase modifications are created, maintained, and repaired, and how their spatial distribution impacts genome function, is lacking.

Incorporation of uracil into DNA creates detrimental or beneficial mutations, depending on context. To sustain DNA replication, cells must synthesize or scavenge precursors to accumulate a pool of nucleotide triphosphates. A key step of thymidine triphosphate (TTP) synthesis is catalyzed by thymidylate synthase, which converts dUMP to dTMP using tetrahydrofolate as a methyl donor. One branch of the TTP biosynthetic pathway uses dUTP as an intermediate, which can be incorporated into DNA in the form of A:U base pairs. The upstream production of dUMP is catalyzed by deamination of dCMP by deoxycytidylate deaminase or pyrophosphorolysis of dUTP by the dUTP pyrophosphatase (Dut1). Dut1 is essential for viability and normal nucleotide metabolism: In the absence of Dut1, cells simultaneously accumulate dUTP and deplete TTP pools (Gadsden et al. 1993), causing a futile cycle of uracil incorporation and repair that leads to extensive DNA damage (Kavli et al. 2007).

Uracil in DNA is removed by uracil DNA glycosylase (UDG) enzymes, which scan double-stranded DNA for uracil and cleave its glycosidic bond (Krokan et al. 2002; Kavli et al. 2007). Simultaneous inactivation of dUTPase and uracil DNA glycosylase in *E. coli* results in viable cells that accumulate significant amounts of uracil in their DNA due to increased dUTP levels and an inability to remove uracil from DNA (Warner et al. 1981). Mutation of the dUTPase and uracil DNA glycosylase genes has also been used to cause uracil incorporation in *S. cerevisiae* (Guillet et al. 2006) and *C. elegans* (Dengg et al. 2006). Regions of high transcription exhibit elevated dUTP incorporation, suggesting that levels of UTP needed to sustain transcription can be converted to dUTP for incorporation into DNA (Kim and Jinks-Robertson 2009).

Pharmacologic treatments and metabolic imbalances also promote dUTP incorporation into DNA. Thymidylate synthase is a major target of anti-metabolite drugs, such as pemetrexed and 5-fluorouracil, which inhibit TTP production by thymidylate synthase and simultaneously increase dUMP and dUTP levels (Longley et al. 2003). Because thymidylate synthase uses tetrahydrofolate as a methyl donor, folate deficiency also alters the pool of available nucleoside precursors for TTP synthesis, and phenocopies anti-folate therapy by increasing the cellular levels of dUTP, causing its incorporation into DNA and subsequent chromosome instability (Blount et al. 1997).

Uracil in DNA also plays a prominent role in adaptive immunity. In B cells, somatic hypermutation and class switch recombination are mediated by the deamination of cytosine residues to uracil by the activation-induced cytosine deaminase (AICDA, also known as AID). AID is recruited to immunoglobulin loci and promotes somatic hypermutation by pseudo-randomly deaminating cytosines to uracil, which are repaired by error-prone polymerases, creating diversity (Maul and Gearhart 2010). Aberrant AID localization can promote hypermutation of tumor suppressor genes (Klemm et al. 2009) and initiates chromosome translocations via cytosine deamination and base excision repair. Signatures of uracil-mediated mutations are widespread among cancer subtypes, and dysregulation of APOBEC family cytidine deaminases was implicated as the cause of these mutations (Alexandrov et al. 2013).

DNA exposure to ultraviolet light creates adducts like cyclobutane dimers and 6-4 photoproducts, formed between adjacent pyrimidines (Pfeifer 1997). Some organisms encode photolyase enzymes that recognize pyrimidine dimers and convert them to “monopyrimidines” in an FAD-dependent reaction using visible light. In the absence of photolyase repair, pyrimidine dimers are excised by nucleotide excision repair enzymes, some of which are mutated in human diseases such as xeroderma pigmentosum, in which UV light creates damage that cannot be repaired (Friedberg 2001). In addition, some translesion DNA polymerases are capable of incorporation across from a pyrimidine dimer in the template strand (Sale 2013).

Pyrimidine dimer formation and prevalence in DNA is dictated by several factors including CpG methylation status, dinucleotide content, and chromatin context (Smerdon and Conconi 1999; Law et al. 2013; Zavala et al. 2014). Moreover, repair of UV photoproducts is strongly influenced by genomic context. Photoproducts formed on transcribed template strands are repaired much more efficiently than nontemplate strands by transcription-coupled repair (Hanawalt and Spivak 2008). In addition, some hot spots of pyrimidine dimer formation are repaired efficiently, whereas other sites are more likely to yield mutations (Tornaletti et al. 1994; You et al. 2000). However, studies of pyrimidine dimer formation in the human genome have been restricted to specific loci (Pfeifer et al. 1991; Törmänen and Pfeifer 1992) or indirectly monitor pyrimidine dimer repair on transfected DNA substrates (Protić-Sabljić et al. 1986), yielding a narrow view of pyrimidine dimer formation and repair in large genomes.

Some methods provide information about specific sites of modified bases in a genome, but have limited applicability because of the need to interrogate a region of interest by PCR. For example, ligation-mediated PCR (LM-PCR) maps nucleobase modifications by ligating PCR-competent adaptors to the ends of cDNA created by termination of polymerase elongation in vitro when it encounters nicks and single base gaps introduced by DNA modification (Sugasawa 2010; Yan et al. 2011; Besaratinia and Pfeifer 2012). LM-PCR has been used to map UV modification using base excision repair enzymes coupled with gene specific probes (Sugasawa 2010; Yan et al. 2011; Besaratinia and Pfeifer 2012) and has also been used to map uracil in the context of AID-mediated deamination (Maul et al. 2011).

Other methods provide global views of nucleobase modification but suffer from low resolution or scalability. A number of methods have been developed to examine DNA methylation and demethylation by chemical modification or affinity purification of 5-methylcytosine and 5-hydroxymethylcytosine (Wu and Zhang 2014). These methods can provide single-nucleotide resolution measures of DNA methylation and demethylation, but they have not been widely applied due to the cost of whole genome coverage needed for accurate determination of sites of chemical modification. Microarray approaches have been used to survey DNA damage caused by ultraviolet light and methylation throughout the genome, using antibodies with an affinity for specific modified nucleobases or enzymes that recognize and cleave modification nucleobases (Schumacher et al. 2006; Zavala et al. 2014). These methods achieve resolution in the 100–1000 base pair range, precluding direct identification of specific sites of DNA modification. A new method based on ChIP-seq for mapping sites of single-stranded DNA enables global studies of putative DNA damage but does not identify specific modified nucleobases (Zhou et al. 2013). Finally, new single-molecule sequencing platforms detect a variety of modified nucleobases in their native contexts (Clarke et al. 2009; Clark et al. 2011; Kozdon et al. 2013), but nanopore sequencers are not yet widely available, and real-time analysis of single molecule polymerase incorporation events suffers from a high error rate and high cost of genome-wide coverage, limiting comprehensive characterization of large eukaryotic genomes.

Here we developed a method that couples the specificity and efficiency of excision repair enzymes with the scale and throughput of massively parallel DNA sequencing to identify single sites or local content of modified nucleobases throughout the genome. This new Excision-seq method provides advantages over existing methods by generating sequencing libraries that are enriched for sites of modification, while maintaining high resolution mapping information. The Excision-seq method is applicable to the detection of DNA modifications for which cognate repair enzymes are available.

## Results

### Development of Excision-seq

**Figure 1.**
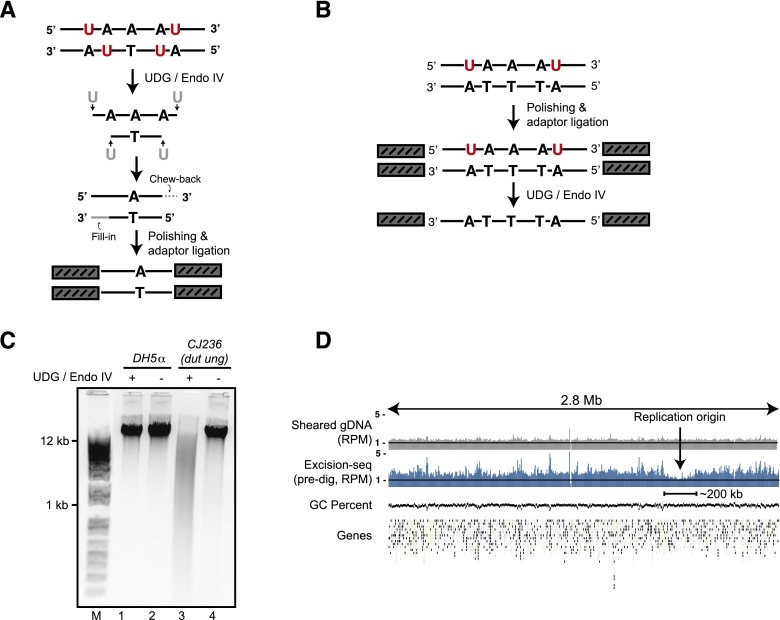
Excision-seq methods for mapping modified nucleobases in genomic DNA. (*A*) In “predigestion” Excision-seq for uracil, uracil-containing DNA is cut with a base excision repair enzyme (e.g., *E. coli* UDG, red). Released fragments are end-repaired, A-tailed, ligated to adaptors, and PCR amplified. Sequences derived from this library identify the positions of modified bases (e.g., one base upstream of the 5′-most position of the read). (*B*) In “post-digestion” Excision-seq for uracil, DNA is sheared mechanically, then treated by standard polishing and ligation. A base excision enzyme cleaves one or both strands containing modified bases. Intact strands remaining after digestion are PCR amplified and sequenced. (*C*) Genomic DNA isolated from *dut ung E. coli* is digested by UDG and T4 endonuclease IV (cf. lanes *3* and *4*), whereas genomic DNA isolated from a wild-type strain is not digested (lanes *1* and *2*). (*D*) Normalized coverage from shotgun sequencing of mechanically sheared genomic DNA (gray, reads per million [RPM]) and predigestion Excision-seq for uracil (blue, RPM) for a 2.8-Mb region of the *E. coli* chromosome. GC-content and the positions of protein-coding genes are plotted *below*. Uracil content is lowest in a region centered on the origin of replication, encompassing ∼200 kb of DNA.

We developed methods to map modified nucleobases in genomic DNA with high precision by coupling base and nucleotide excision repair enzymes with next-generation DNA sequencing (Excision-seq). Excision-seq comprises two related approaches that reflect the point at which modified nucleobases are removed: In “predigestion” Excision-seq (Fig. 1A), a base excision repair enzyme removes modified bases in genomic DNA, creating abasic sites that are removed with T4 endonuclease IV, leaving a single base gap. When the modified base is present in high abundance, this treatment releases small double-stranded DNA fragments that can be converted into libraries suitable for next-generation DNA sequencing. Importantly, the ends of these fragments correspond to the previous locations of the modified base, allowing their identification by sequencing. The predigestion approach precisely identifies positions of modified base incorporation. One drawback of predigestion Excision-seq is that high levels of the modified base are required to yield fragments upon digestion for subsequent library construction. To address this limitation, we developed a complementary approach termed “post-digestion” Excision-seq (Fig. 1B), in which genomic DNA is sheared and converted to a sequencing library by end polishing and adaptor ligation. Prior to PCR, the library is treated with a base excision repair enzyme to destroy one or both strands from each double-stranded DNA in the library that contains modified bases. Undigested strands are amplified and sequenced to identify regions in which the modified nucleobases were not present. Post-digestion Excision-seq is useful when modified bases are excluded from regions of the genome or when the abundance of the modified bases is insufficient to yield sufficient double-strand breaks needed for the predigestion method. The post-digestion method provides information about the content of modified nucleobases and can achieve resolution that correlates with fragment lengths used to build the sequencing library.

### Application of Excision-seq to study uracil in DNA

We used predigestion Excision-seq to map the locations of uracil in the *E. coli* genome. We digested genomic DNA from *E. coli* with a hypomorphic dUTPase that was also missing the uracil repair enzyme (Warner et al. 1981) in vitro with uracil DNA glycosylase (UDG) and T4 Endonuclease IV (Fig. 1C), yielding DNA fragments that were converted to a sequencing library. In parallel, we prepared a library from mechanically sheared DNA to test whether uracil content affects library preparation efficiency. We collected and aligned ∼10 million sequences from these libraries and found that the library prepared from genomic DNA exhibited uniform coverage across the genome (Fig. 1D). The low coverage of reads from the Excision-seq library in the ∼200-kb region surrounding the replication origin unexpectedly suggested low levels of uracil incorporation in this region of the genome (Fig. 1D).

**Figure 2.**
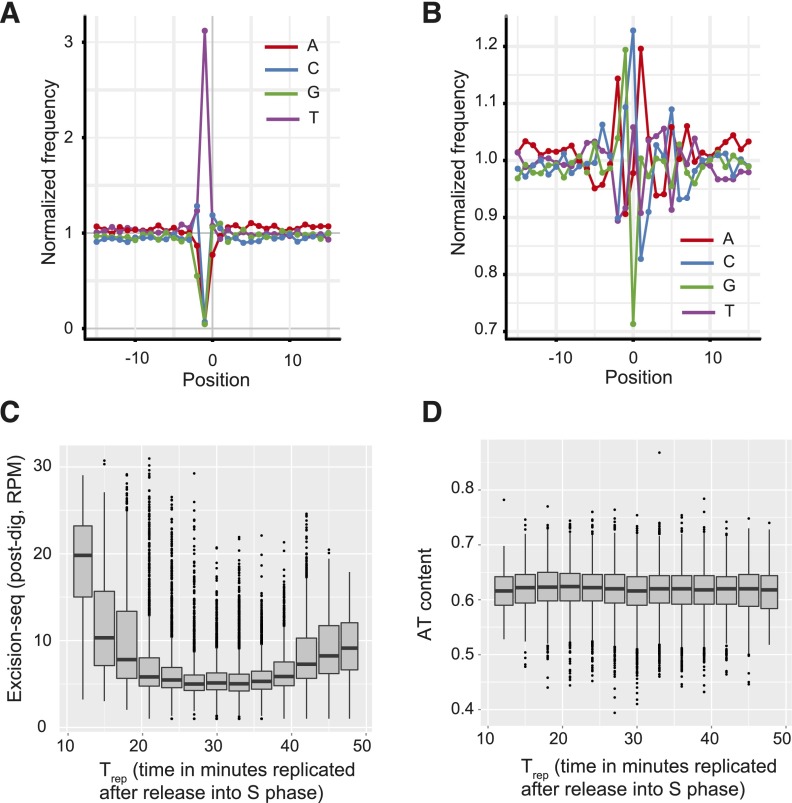
Excision-seq mapping of uracil content in the budding yeast genome. (*A*) Normalized frequency of nucleotides relative to mapped positions of sequences from predigestion Excision-seq libraries for mapping *S. cerevisiae* uracil content. Position 0 corresponds to the mapped position of the 5′ end of 11,326,044 sequencing reads; negative numbers are upstream. Frequencies were normalized to genomic mononucleotide frequencies. (*B*) Normalized frequency of nucleotides relative to mapped positions of sequences from post-digestion Excision-seq libraries for mapping *S. cerevisiae* uracil content. Position 0 corresponds to the mapped position of the 5′ end of 2,939,357 sequencing reads (frequency normalization and colors identical to *A*). (*C*) Boxplot comparing post-digestion Excision-seq mapping of uracil in *S. cerevisiae* to replication timing (T_rep_) (Raghuraman et al. 2001). Mean signals were calculated in 500-bp windows across the genome. (*D*) Boxplot comparing mean genomic AT content in *S. cerevisiae* to replication timing; 500-bp regions are identical to *C*.

To further study this phenomenon, we used pre- and post-digestion Excision-seq to map uracil in a *dut1-1 ung1*∆ budding yeast strain (Guillet et al. 2006). We examined the frequency of bases surrounding the mapped positions of reads in each library. In predigestion Excision-seq libraries for mapping uracil, we expected to find that the base upstream of mapped positions would correspond to a T residue, representing the previous location of a uracil (Fig. 1A). More than 98% of reads from uracil Excision-seq libraries mapped to positions downstream from T residues (Fig. 2A), indicating robust recovery of uracil sites. In post-digestion Excision-seq libraries for uracil, we expected a more random distribution of base identity at the sites of linker ligation (Fig. 1B) and found relatively uniform levels of base identity near the sites of linker ligation (Fig. 2B).

Given the low uracil content at the *E. coli* replication origin, we considered whether uracil content in *S. cerevisiae* would correlate with replication timing. We quantified the relationship between uracil content and replication timing (Raghuraman et al. 2001; Yabuki et al. 2002) by calculating the coverage in post-digestion Excision-seq uracil libraries in 500-bp windows, and compared the coverage to replication timing (Fig. 2C). We found that uracil content was lowest (e.g., highest post-digestion signals) in early-replicating regions. Uracil content increased steadily over the first ∼8 min of S phase and remained constantly high until uracil content decreased again within the last 10 min of S phase. As a control, we also calculated AT content over the same intervals and found no significant variation in AT content across these regions (Fig. 2D), confirming that uracil content is not driven by variation in genomic nucleotide content. In the *E. coli* predigestion Excision-seq mapping of uracil, we identified local correlation between uracil content and local GC content at a size range of 1–10 bp; this bias was not apparent in the *S. cerevisiae* mapping data. Thus, whereas there is some relationship between uracil content and genomic AT content, the variation in uracil content that we observe changes over the kilobase scale, far greater than local changes in AT content.

**Figure 3.**
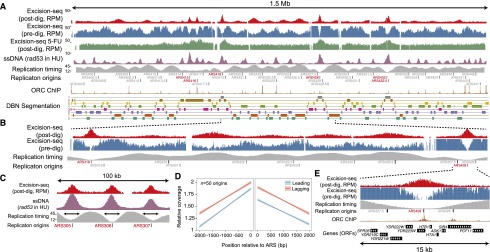
Excision-seq maps of uracil content in the budding yeast genome. (*A*) Data showing the entire yeast chromosome 4 for *dut1-1 ung1*∆ yeast using post-digestion Excision-seq (red, reads per million [RPM]), predigestion Excision-seq (blue, RPM), post-digestion Excision-seq data for *ung1*∆ yeast treated with 5-fluorouracil (5-FU) (green, RPM), single-stranded DNA accumulation caused by hydroxyurea treatment of a *rad53* yeast strain (purple, arbitrary units) (Feng et al. 2006), replication timing data (T_rep_, minutes replicated after G_1_ release) (Raghuraman et al. 2001) (gray), annotated origins of replication (Nieduszynski et al. 2007), ORC chromatin immunoprecipitation signals (Eaton et al. 2010) (brown, coverage), and labeled segments from an eight-state DBN segmentation (Hoffman et al. 2012) incorporating replication timing (Yabuki et al. 2002) and post-digestion Excision-seq mapping of uracil. (*B*) A 450-kb region of chromosome 4 highlights patterns of uracil incorporation in early-replicating origins (*ARS418* and *ARS428*), as well as uracil depletion in late-replicating regions. (*C*) Correspondence of peak widths between post-digestion Excision-seq (red) and ssDNA accumulation (Feng et al. 2006) (purple) at three early-replicating origins in a 100-kb region of chromosome 3. (*D*) Post-digestion Excision-seq measurement of uracil content for 50 early-replicating origins. Lagging strands have ∼1.3-fold higher relative coverage than leading strands in post-digestion Excision-seq data, reflecting increased uracil content in leading strands. (*E*) A 15-kb region of chromosome 4 highlights patterns of uracil incorporation at the early-replicating origin *ARS428*.

Visualization of Excision-seq mapping data for uracil at the chromosomal level reinforced a correlation between replication timing and uracil content. A total of 42 early-firing origins were depleted of uracil, (e.g., *ARS418* and *ARS428*) (Fig. 3A–C,E). In addition, late-replicating regions exhibit a significant but more modest depletion of uracil (Fig. 3A,B). Sequence read coverage in post-digestion Excision-seq data is inversely proportional to content of the modified nucleobase: Regions with high levels of coverage have low levels of the modified base and vice versa. The mean coverage of post-digestion Excision-seq data for mapping uracil in *dut1-1 ung1∆* yeast was 8.2 reads per base, with a standard deviation of 6.8. With respect to replication timing, there was a 15-fold difference in Excision-seq data coverage between early-firing origins and other regions ∼10 kb away (e.g., origin *ARS428*) (Fig. 3E). In contrast, in late-replicating regions, there was a sevenfold difference between the latest replicating DNA and nearby regions (e.g., peaks between ARS406 and 409) (Fig. 3A). Because post-digestion Excision-seq maintains strand information (Fig. 1B), we examined uracil content in leading and lagging strands. We selected 50 early-firing origins (Raghuraman et al. 2001) and examined the uracil content of leading and lagging strands within 2 kb of the ARS consensus sequence. We found that sequence coverage of the lagging strand in post-digestion Excision-seq data was ∼1.3-fold higher than the leading strand, suggesting a bias toward higher uracil incorporation in the leading strand relative to the lagging strand during early replication (Fig. 3D) and possibly implicating a bias in uracil incorporation by DNA polymerases or pool availability.

We used Excision-seq to map uracil content in *ung1*∆ yeast during pharmacological inhibition of thymidylate synthase with 5-fluorouracil (5-FU) (Seiple et al. 2006). Genomic DNA from *ung1*∆ yeast treated with 5-FU had higher levels of overall uracil incorporation (Fig. 3A, green) but showed uracil depletion at early-firing origins of replication (Fig. 3A, *ARS418* and *ARS428*), suggesting that early thymidylate synthesis is less sensitive to 5-FU treatment. The lack of uracil incorporation that we observed in late-replicating regions of the genome (Fig. 3A, red and blue) was not present in these data, suggesting that 5-FU might activate a checkpoint, preventing late origins from firing (Santocanale and Diffley 1998; Feng et al. 2006; Seiple et al. 2006).

To discern global features of uracil Excision-seq data, we used a previously developed segmentation approach based on dynamic Bayesian networks (Hoffman et al. 2012) to identify correlations between uracil incorporation and other functional features of the genome. We segmented the yeast genome using uracil Excision-seq data and replication timing data (Raghuraman et al. 2001) to identify coherent patterns between these data sets (Fig. 3A). This analysis identified two major classes of genomic regions showing uracil depletion: those that replicate earliest and latest. At early-firing origins (e.g., Fig. 3A, *ARS428* and label 7 in the segmentation), uracil content is low, centered on the site of Orc1 binding (Eaton et al. 2010), and uracil levels slowly increase at sites upstream of and downstream from the origin, similar to the phenomenon observed in *E. coli* (Fig. 1D). However, the latest replicating regions of the chromosome also exhibit uracil depletion (Fig. 3A, label 1 in the segmentation): The region between two early-firing origins (*ARS418* and *ARS428*) exhibits variation in uracil incorporation that correlates with late-replicating regions. However, despite uracil depletion in late-replicating regions, the extent of depletion is reduced relative to early-firing origins (Fig. 3B, cf. peaks over *ARS418* and *ARS428* with intervening, late-replicating regions). To identify other genomic features that might correlate with genomic uracil content, we performed other segmentations, including DNase I hypersensitivity mapping data (Hesselberth et al. 2009) and mRNA expression levels measured by RNA-seq (Levin et al. 2010), but found that these signals did not qualitatively change the segmentations (data not shown), emphasizing that genomic uracil content correlates most strongly to replication timing.

### Application of Excision-seq to study pyrimidine dimers in DNA

**Figure 4.**
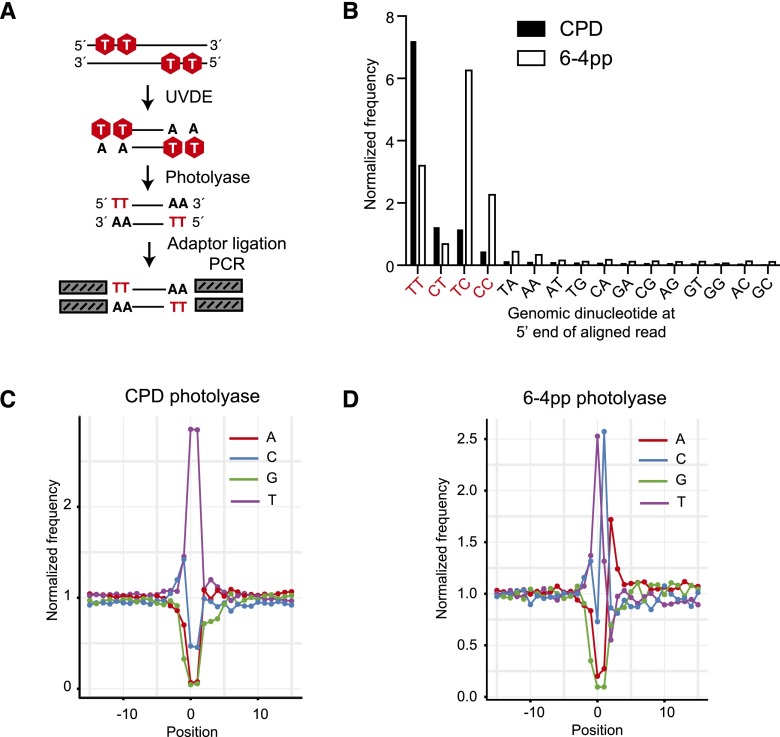
Excision-seq mapping of dipyrimidines in the budding yeast genome. (*A*) In predigestion Excision-seq for pyrimidine dimers, UV-damaged DNA is cleaved with UVDE, releasing double-stranded fragments with five dipyrimidines (red). Fragments are treated with CPD or 6-4pp photolyase enzymes, repairing dipyrimidines to “mono” pyrimidines, and yielding ends compatible with polishing, ligation, and PCR. (*B*) Analysis of sequencing libraries treated with CPD or 6-4pp photolyase prepared from UV-irradiated DNA showed an enrichment of sequence reads with dipyrimidine ends (red text) relative to genomic dinucleotide content and recapitulated the known specificity of the photolyase enzymes (Chowdhury and Guengerich 2008). (*C*) Frequency of nucleotides relative to mapped positions of sequences from predigestion Excision-seq libraries for mapping cyclobutane dimers in *S. cerevisiae*. Position 0 corresponds to the mapped position of the 5′ end of 5,063,196 sequencing reads. (*D*) Frequency of nucleotides relative to mapped positions of sequences from predigestion Excision-seq libraries for mapping 6-4 photoproducts in *S. cerevisiae*. Position 0 corresponds to the mapped position of the 5′ end of 3,655,251 sequencing reads.

We extended Excision-seq to map two main classes of dipyrimidines caused by exposure to UV light: cyclobutane pyrimidine dimers (CPDs) and 6-4 photoproducts (6-4pp) (Pfeifer 1997). We collected high molecular weight genomic DNA from cells irradiated with UV light (∼10,000 J/m^2^; <10% cell viability) and treated damaged DNA with *S. pombe* Uve1 (also known as UVDE), which recognizes and cleaves upstream of both CPDs and 6-4pp (Fig. 4A; Avery et al. 1999). Pyrimidine dimers created at the ends of DNA fragments upon UVDE digestion inhibited downstream polishing and adaptor ligation (data not shown), so we repaired 5′ pyrimidine dimers in vitro to “mono” pyrimidines using photolyase enzymes that recognize either cyclobutane dimers or 6-4 photoproducts (Fig. 4A; Sancar and Sancar 2006). These repaired 5′-dipyrimidine ends are compatible with subsequent polishing, adaptor ligation, and PCR. DNA sequences from these libraries were collected and aligned to the budding yeast genome to determine the dipyrimidine at their 5′ ends. More than 85% of the aligned sequences acquired from libraries prepared by treatment with CPD and 6-4pp photolyases derived from genomic positions with pyrimidine dimers—validating the method. In total, we identified 1,249,684 sites of CPD formation (38% of all genomic dipyrimidines; 72% of TT) and 107,490 sites of 6-4pp formation (5% of all genomic dipyrimidines). Dipyrimidine content of sequences reflected known photolyase specificities, recapitulating the expected distribution of CPDs (i.e., cyclobutane dimer prevalence is greatest for TT dipyrimidines, followed by TC, CT, and CC) and 6-4 photoproducts (i.e., TC dinucleotides are most abundant, followed by TT, CC, and CT) (Fig. 4B; Douki and Cadet 2001).

We examined local base content near the sites of linker attachment in the CPD and 6-4pp Excision-seq libraries (Fig. 4C,D). In the CPD library, 60% of the reads began with TT dinucleotides, followed by CT dinucleotides (15% of reads) (Fig. 4B). In the CPD library, normalized nucleotide frequencies upstream of and downstream from the first and second positions were not skewed relative to genomic frequencies of the budding yeast genome (Fig. 4C). For the 6-4pp libraries, the first and second positions of the reads exhibited a strong bias toward pyrimidines. Notably, the base downstream from the dipyrimidine in the 6-4pp libraries was most often an A residue (with TCA and TTA comprising ∼20% of the total reads), suggesting that 6-4 photoproducts are preferentially created at these trinucleotides, or that the *X. laevis* 6-4 photolyase enzyme preferentially repairs these sites. This bias is not likely due to the UVDE enzyme, as it was also used to prepare the CPD libraries, which did not have detectable bias toward any residue at the position downstream from the dipyrimidine (Fig. 4D).

## Discussion

Application of Excision-seq to study uracil content in DNA revealed previously unknown variation in uracil content that is highly correlated with DNA replicating timing. We hypothesize that variation in uracil content is established by changes in dNTP pool composition such that the pool of nucleotides available for early and late replication contains higher levels of TTP than dUTP. Comparison of our data to previous studies that measured the levels of single-stranded DNA accumulation following depletion of dNTPs with hydroxyurea (HU) (Feng et al. 2006) revealed that the regions of depleted uracil content (e.g., post-digestion Excision-seq peaks) are remarkably similar to the amount of DNA replicated from early-firing origins in the presence of HU (Fig. 3C). This correlation suggests that these two phenomena—dNTP depletion and TTP:dUTP equilibration—may happen at a similar time during S phase. Recent studies also showed that dNTP pools synthesized during G_1_/S are limiting for DNA synthesis, allowing ∼5 kb of DNA to be replicated before a critical transition during replication (Poli et al. 2012). Together, these and our data suggest that dNTPs made during G_1_ (Koç et al. 2004) may be compositionally pure of dUTP, and the transition from low to high uracil content (e.g., summit to shoulder of Excision-seq peaks) (Fig. 3C) reflects equilibration of dUTP and TTP levels. Notably, the phenomenon of uracil content variation may enable simplified mapping of replication timing, as *ung1∆* yeast strains are readily created and human cell lines expressing a bacteriophage uracil DNA glycosylase inhibitor have undetectable levels of nuclear human UNG (also known as UNG1 and UNG2) activity (Weil et al. 2013).

The depletion of uracil in late-replicating regions found in uracil Excision-seq data is unexpected and suggests that dUTP availability is also reduced toward the end of replication. The dUMP substrate of thymidylate synthase is produced either by conversion of dUTP to dUMP by dUTPase or by conversion of dCMP to dUMP by deoxycytidylate deaminase (Dcd1). We speculate that the TTP:dUTP ratio is high during early and late replication due to limited production of dUTP (e.g., less ribonucleotide reductase-mediated reduction of UDP), while TTP levels are maintained by the activity of dCMP deaminase.

One prediction of uracil content variation is that the mutational signature of uracil incorporation might correlate with DNA replication timing, possibly over evolutionary time scales. In budding yeast, Ung1 action on A:U base pairs produces abasic sites in the template strand that are copied by the error-prone Rev1/Rev3 translesion polymerase, which incorporates a C across from the abasic site, yielding an A-to-C transversion (Collura et al. 2012). However, these diagnostic A:C transversions were not correlated with replication timing in yeast (Agier and Fischer 2012) or humans (Stamatoyannopoulos et al. 2009), suggesting that uracil excision repair is highly efficient under physiological conditions, limiting uracil-mediated mutational signatures.

Variation in uracil content protects large chromosomal regions containing replication origins from uracil incorporation. If a similar mechanism for uracil content variation operates in human cells, it might inherently counteract common chemotherapies such as 5-fluorouracil, which raise dUTP levels to promote cycles of incorporation and repair, causing DNA damage and apoptosis. Elucidation of the mechanism underlying uracil content variation may therefore have implications for the design and delivery of therapeutics that cause nucleotide pool imbalances to promote cell death.

Unlike uracil incorporation, CPD and 6-4pp dimers were uniformly distributed across the genome, consistent with previous studies showing uniform densities of CPD formation (Teng et al. 2011). Future application of Excision-seq to map UV photoproducts may uncover more subtle patterns associated with genomic features, including nucleosome positions or other structural features. These studies could provide insight into the nature of mutational hotspots found in DNA preferentially caused by 6-4 photoproducts, which are more mutagenic than CPD photoproducts (Mitchell 1988). Application of Excision-seq in mammalian cells would enable the study of photoproduct formation in the context of methylated CpG dinucleotides, which are prone to mutation (You et al. 1999).

Analysis of Excision-seq signals for the mapping of uracil and pyrimidine dimers enables an estimation of the specificity of the Excision-seq method. Nearly 98% of the reads from the uracil predigestion libraries map to positions downstream from a T residue (i.e., uracil in the DNA) (Fig. 2A), demonstrating the high specificity of UDG and consistent with previous measurement of its specificity for uracil in vitro (Stivers et al. 1999). Similar measurements of the UVDE enzyme show that it efficiently recognizes and cleaves at pyrimidine dimers but also recognizes a variety of other nucleobase lesions in DNA (Avery et al. 1999). The specificity for dipyrimidines is recapitulated in the CPD and 6-4pp Excision-seq libraries, where many of the reads begin with dipyrimidines (Fig. 4B). These data suggest that the specificity of Excision-seq is largely dictated by the specificity of excision repair enzymes. Based on its recovery of expected sites of modification, Excision-seq has a low empirical false positive rate for individual sites of uracil modification (2% for uracil predigestion libraries). For the CPD and 6-4pp libraries, 93% and 86% of the sequences began with dipyrimidines, initially suggesting a high false positive rate (7% and 14%, respectively). However, signals from the CPD and 6-4pp libraries could also be attributed to the extended ability of UVDE to cleave at other types of bulky DNA lesions (Avery et al. 1999). Notably, AA and TA were the most abundant dinucleotides other than dipyrimidines in both CPD and 6-4pp libraries, with AA dinucleotides at the beginning of 4% of the reads for both libraries and TA dinucleotides beginning 3% of reads for CPD libraries. Little is known about photoproducts involving purines (Pfeifer 1997), but previous studies detected photoproduct formation in synthetic TA and AA dinucleotides (Bose et al. 1983; Gallagher and Duker 1986), consistent with signals we observe from photoproducts formed in genomic DNA. It is possible that the AA and TA dinucleotides present in the CPD and 6-4pp libraries more accurately reflect the UV-induced formation of photoproducts at genomic dinucleotides involving purines, or these could be false positive signals generated by, e.g., photolyase bias in the assay. Finally, other factors including the quality of DNA starting material for Excision-seq libraries could influence the false positive rate for individual samples. For example, predigestion Excision-seq libraries should be constructed with carefully prepared high molecular weight DNA to reduce the amount of nonspecific fragmentation, which would be captured in a sequencing library (Fig. 1A).

The sensitivity of the Excision-seq method can also be considered. In predigestion Excision-seq, the ability to capture low levels of modified nucleobases is influenced by the efficiency of the excision repair enzyme and sufficient levels of modified nucleobases to enable creation of double-stranded DNA fragments. We found that shearing of DNA from *dut1-1 ung1∆* yeast by UDG was not enhanced by extended incubation times (data not shown), indicating quantitative cleavage of uracil bases. The sensitivity of post-digestion Excision-seq is dictated by the overall incorporation levels of modified nucleobases at the same position in a population of molecules. In the future, the combination of post-digestion Excision-seq with single molecule tagging strategies should enable more precise quantitation of modified nucleobase incorporation in large genomes (Schmitt et al. 2012; Hiatt et al. 2013).

The availability of a variety of excision repair enzymes will facilitate the study of other modifications by Excision-seq. Many cell intrinsic (e.g., inosine) and extrinsic (e.g., 8-oxo-guanine) modified nucleobases have cognate repair enzymes, possibly enabling their study by Excision-seq (Tchou et al. 1994; Saparbaev et al. 2000). The removal of oxidized cytosine bases during DNA demethylation is catalyzed by the base excision repair enzyme TDG, which could be used in Excision-seq to generate maps of oxidized cytosine bases in mammalian DNA (Kohli and Zhang 2013). Finally, ribonucleotide incorporation into DNA could also be studied by Excision-seq using Ribonuclease HII enzymes to cleave at sites of ribonucleotide incorporation (Nick McElhinny et al. 2010).

Excision-seq enables the analysis of primary DNA modification events independently from fixed sequence polymorphisms, facilitating study of the mutational process. Large-scale surveys of cancer genomes have identified mutational signatures attributable to environmental exposure or dysregulated cellular physiology (Alexandrov et al. 2013). The Excision-seq method will be useful in generating high resolution global maps of DNA modification that can be integrated with surveys of DNA sequence polymorphisms (The 1000 Genomes Project Consortium 2012) and functional chromatin states (The ENCODE Project Consortium 2012) to provide insight into how DNA modifications are formed, how repair processes facilitate their conversion to mutation, and how chromatin context influences both DNA modification and repair.

## Methods

### Strains and oligonucleotides

Strains and oligonucleotides are available in Supplemental Methods.

### Excision-seq library methods

Detailed methods for constructing Excision-seq libraries are available in Supplemental Methods. Briefly, in predigestion Excision-seq, DNA containing modified nucleobases was treated with excision repair enzymes, and adaptors were ligated to the sites of cleavage, facilitating PCR amplification of these fragments. In post-digestion Excision-seq, libraries of mechanically sheared genomic DNA were treated with excision repair enzymes to destroy strands containing the modified base, preventing their PCR amplification. Libraries were sequenced on Illumina MiSeq or HiSeq 2000 platforms using standard protocols.

### Analysis of Excision-seq data

Sequences were analyzed by alignment to a reference genome (*sacCer1*) using Bowtie 2 (Langmead et al. 2009) and SAMtools (Li et al. 2009), processed to bedGraph format using BEDTools (Quinlan and Hall 2010), and visualized in the UCSC Genome Browser (Karolchik et al. 2011). Coverage at each position was normalized by the number of reads aligned in the library (i.e., reads per million [RPM]). Using this method, the level of coverage at a specific site or region in the genome represents the relative quantity of modified base at that position. For Excision-seq libraries mapping dipyrimidines, dinucleotide counts for the 5′ ends of the reads were determined, and the frequencies of dinucleotide combinations were normalized to background frequencies found in *S. cerevisiae* genomic DNA to account for the A:T bias in the genome. Software and pipelines used to analyze data are available on GitHub (https://github.com/hesselberthlab/modmap).

### DBN segmentation of genomic data sets

We applied a segmentation approach using dynamic Bayesian networks (Hoffman et al. 2012) to find correlations between uracil content and replication timing (Raghuraman et al. 2001; Yabuki et al. 2002). An eight-state model was trained on 1% of the genome using a resolution of 500 bp, and the parameterized model was used to decode the rest of the genome.

## Data access

Raw and processed sequencing data (FASTQ and bedGraph formats) from this study have been submitted to the NCBI Gene Expression Omnibus (GEO; http://www.ncbi.nlm.nih.gov/geo/) (Barrett et al. 2010) under accession number GSE51361.
